# Pregnancy outcomes and newborn characteristics in women with follicular fluid thyroid autoantibodies undergoing assisted reproduction

**DOI:** 10.5937/jomb0-35243

**Published:** 2023-01-20

**Authors:** Sanja Medenica, Eliana Garalejić, Džihan Abazović, Zoran Bukumirić, Stavroula A. Paschou, Biljana Arsić, Snežana Vujošević, Biljana Međo, Miloš Žarković

**Affiliations:** 1 University of Montenegro, School of Medicine, Clinical Center of Montenegro, Internal Medicine Clinic, Department of Endocrinology, Podgorica, Montenegro; 2 Clinic for Gynecology and Obstetrics "Narodni front", In Vitro Fertilisation Department, Belgrade; 3 University of Belgrade, School of Medicine, Belgrade; 4 Emergency Medicine Center of Montenegro, Podgorica, Montenegro; 5 University of Belgrade, Institute for Medical Statistics and Informatics, Faculty of Medicine, Belgrade; 6 National and Kapodistrian University of Athens, School of Medicine, Alexandra Hospital, Endocrine Unit and Diabetes Centre, Department of Clinical Therapeutics, Athens, Greece; 7 University Children's Hospital, Pediatric Intensive Care Unit, Belgrade; 8 Clinical Center of Serbia, Clinic of Endocrinology, Diabetes and Metabolic Diseases, Department of Thyroid Gland Disease, Belgrade

**Keywords:** assisted reproductive technology, follicular fluid, pregnancy, thyroid autoimmunity, asistirana reproduktivna tehnologija, folikularna tečnost, trudnoća, tiroidna autoimunost

## Abstract

**Background:**

Higher levels of thyroid autoantibodies in follicular fluid (FF) of thyroid autoimmunity (TAI) positive women are strongly correlated with serum levels and may have effect on the post-implantation embryo development. Literature highlights that levothyroxine (LT4) treatment may attenuate the risk of adverse pregnancy outcomes. The aim of the study was to estimate the pregnancy and newborn outcomes in women with FF thyroid autoantibodies undergoing assisted reproductive technology (ART).

**Methods:**

The study population included 24 women with confirmed clinical pregnancy, 8 TAI positive and 16 TAI negative women. LT4 supplementation was applied in 20.8% patients, TAI positive.

**Results:**

Pregnancy outcomes were: twin pregnancy rate 41.7%, early miscarriage rate 8.3%, late miscarriage rate 4.2%, preterm birth rate 16.7%, term birth rate 70.8%, live birth rate 96.0%. There was significant difference in serum and in FF TgAbs (p< 0.001)between the groups according to TAI, while serum fT_3_ was lower in the group with TAI (p = 0.047). Serum P_4_ was higher in LT4 treated group (p = 0.005), with TAI, and newborns in this group had higher birth weight (p = 0.001) and height (p = 0.008). Maternal complications occurred in 23.8% of patients. No congenital malformations in newborns were noted.

**Conclusions:**

Thyroid autoantibodies present in FF may have an effect on the post-implantation embryo development, but have no effect on further course of pregnancy. The special benefit of LT4 treatment for successful ART outcome was demonstrated for newborn anthropometric parameters.

## Abstract (sr)

**Uvod:**

Povišeni nivoi tiroidnih autoantitela u folikularnoj tečnosti (FF) kod žena sa tiroidnom autoimunošću (TAI) nalaze se u snažnoj korelaciji sa nivoima istih u serumu i mogu uticati na postimplantacioni razvoj embriona. Podaci iz literature govore o značaju primene levotiroksina (LT4) u smislu smanjenja rizika od neželjenih ishoda trudnoće. Cilj studije je bio da se proceni ishod trudnoće i karakteristike novorođenčeta žena sa tiroidnim autoantitelima u FF u postupku asistirane reprodukcije (A RT).

**Metode:**

Ispitivana populacija je uključila 24 žene sa potvrđenom kliničkom trudnoćom, 8 TAI pozitivnih i 16 TAI negativnih žena. LT4 suplementacija je primenjena kod 20,8% pacijentkinja, pozitivnih na TAI.

**Rezultati:**

Ishodi trudnoće bili su: stopa blizanačke trudnoće 4 1 ,7 % , stopa ranih sponatnih pobačaja 8,3% , stopa kasnih sponatnih pobačaja 4 ,2 % , stopa prevremenog porođaja 16,7% , stopa terminskog porođaja 70,8% , stopa živorođenosti 96,0% . Postojala je značajna razlika u vrednostima antitela na Tg u serumu i u FF (p < 0 ,0 0 1 ) između grupa shodno TAI, dok je serumski fT3 bio niži u grupi sa TAI (p = 0 ,0 4 7 ). Serumski fT 4 je bio viši u grupi koja je primala terapiju sa LT4 (p = 0 ,0 0 5 ), sa TAI, a novorođenčad u ovoj grupi imala su veću porođajnu težinu (p = 0,001) i dužinu (p = 0 ,0 0 8 ). Maternalne komplikacije bile su prisutne kod 23,8% pacijentkinja. Nisu zabeležene urođene malform acije kod novorođenčadi.

**Zaključak:**

Tiroidna autoantitela u FF mogu da utiču na postimplantacioni razvoj embriona, ali nemaju uticaja na dalji tok trudnoće. Posebna korist terapije LT4 na uspešan ishod ART-a je pokazana za antropometrijske parametre novorođenčeta.

## Introduction

Beside the fact that thyroid autoimmunity (TAI) presence is related to lower chances of achieving pregnancy, there is an association with adverse pregnancy outcomes in both, spontaneously and pregnancies conceived by assisted reproductive technologies (ART) [1]. Subclinical hypothyroidism (SCH) in TAI may remain asymptomatic or even undiagnosed [2] with consequent deterioration of thyroid function to overt hypothyroidism. In women with TAI undergoing *in vitro* fertilization (IVF), controlled ovarian hyperstimulation (COH), even more aggravates thyroid function [2]. The highest levels of thyrotropin (TSH) were demonstrated in the first week after induction of ovulation [3]. The rise could be due to fact that serum estradiol levels during ART reach those seen during the second trimester of pregnancy [4].

Literature highlights that levothyroxine (LT4) treatment may attenuate the risk of adverse pregnancy outcomes [5]. In American Thyroid Association 2017. guideline states that insufficient evidence exists to determine whether LT4 therapy improves the success of pregnancy following ART in TPOAb-positive euthyroid women, but administration of LT4 to TPOAb-positive euthyroid women undergoing ART may be considered given its potential benefits in comparison to its minimal risk [6]. A Chinese research group conducted a population-based trail to reevaluate whether TAI positive infertile women with normal thyroid function would benefit from LT4 treatment with respect to pregnancy outcomes following IVF, but no differences in the clinical pregnancy, live birth and miscarriage rates between groups were observed [7].

We demonstrated the presence of thyroid autoantibodies in follicular fluid (FF) in women with TAI, noting those are not generated in the FF, but cross from the blood. Among fifty-two patients undergoing ART enrolled in our study, pregnancies rates count both per initiated cycle and per embryo transfer (ET) cycle were significantly different between TAI positive and TAI negative group [8]. It was confirmed once again that TAI positive women had less chance to achieve fertility, but with no proven direct impact of TAI on oocytes and embryos during ART treatment [8]. Even though the effect on fertilization and implanation rate should not be neglected considering published data [9]. As suggested in abovementioned study, higher levels of thyroid autoantibodies in FF of TAI positive women are strongly correlated with serum levels and may have effect on the post-implantation embryo development.

Hence, in order to estimate the pregnancy and newborn outcomes in women with FF thyroid autoantibodies undergoing ART, we established primary aim of the study as live birth rate, and secondary aims: late miscarriage rate, preterm and term birth rate, gestational length, delivery method, newborn gender, newborn weight and height.

## Materials and methods

This prospective study included 24 women as study population in whom clinical pregnancy was confirmed. Initially 52 women, 26 TAI positive and the same number of TAI negative (age and body mass index, BMI, matched) underwent ART, in study conducted during the period from November 2014 to July 2016, in Clinic for Gynecology and Obstetrics »Narodni front«, Belgrade, Serbia. Among 26 TAI positive patients in 8 of them clinical pregnancy was confirmed, and in 16 patients of TAI negative group [8]. Ethical committee of Faculty of Medicine, University of Belgrade and Ethical committee of Clinic for Gynecology and Obstetrics »Narodni front«, granted approval for the study and written informed consents were obtained from all subjects.

Confirmed pregnancy was the only inclusion criteria for this study, but all these patients when underwent ART procedure had to fulfill the criteria predefined by the National Expert Commission of the Ministry of Health for biomedical assisted fertilization procedures [10].

### Clinical methodology

Before the initiation of protocol for the COH, TSH, free triiodothyronine (fT_3_), free thyroxine (fT_4_), thyroid peroxidase antibodies (TPOAbs) and thyroglobulin antibodies (TgAbs) levels were measured in serum by Cobas 6000 ROCHE, using *immunochemiluminescent *method. Referent values for the analyses were: TSH 0.40–4.00 mIU/mL, fT_4_ 12.00–22.00 pmol/L, fT_3_ 3.10–6.80 pmol/L, TgAbs 0.0–25.3 IU/mL, upper limit of TPOAbs reference range 19 IU/mL (95 % CI 17–26 IU/mL), adapted for the local population in Serbia [11].

In the following text the ART protocol is described [8]. Two protocols, long gonadotropin-releasinghormone agonist (GnRH-a), (Dipherelin 0,1 mg/mL, Triptorelin, Pharma Swiss, Belgrade, Serbia) or short GnRH-antagonist (GnRH-ant) protocol (Cetrotide 0.25 mg/mL, Cetrorelix acetate, Merck Serono, Frankfurt, Germany), in combination with urinary HMG-a (Menopur 75 i.j., menotrophin-human menopausal gonadotropin HMG, Ferring Pharmaceuticals, Fering B.V. Germany) and/or a recombinant follicle-stimulating hormone FSH (Gonal-F 75 i.j., follitropin alpha, Merck Serono, Modugno, Italy and/or Puregon 50 IU and 100 IU follitropin beta, Merck Sharp & Dohme, Belgrade, Serbia) were used for COH. Final oocyte maturation by 10 000 IU human horionic gonadotropin hCG (Pregnyl, Chorionic gonadotropin, Merck Sharp & Dohme, Belgrade, Serbia) or Ovitrelle 250 micrograms (choriogonadotropin alfa, Merck Serono SpA Modugno, Italy) was done when 3 or more follicles larger than 17 mm in diameter were detected with adequate estradiol (E2) level. Then, 35h hours later the oocyte retrieval was done. We obtained FF from follicles with a diameter 17 mm, and TSH, fT_4_, TPOAbs, TgAbs and progesterone (P4) levels were measured, by automatic analyzer Cobas 6000 ROCHE, using commercial tests of the same company based on the principleof *immunochemiluminescent *method. No specific cutoffs are known for these parameters in FF. ET was carried out second or third day after the oocyte aspiration. For the luteal support P4 was administered.

### Analyzed data

We analyzed late miscarriage defined as spontaneous loss of pregnancy from the 12^th^ to the 20^th ^week of gestation; preterm birth defined by the birth of a live-born or still-born newborn in the period after 20 weeks of gestation, and before 37 completed weeks of gestation; term birth defined as live-born or stillborn-born newborn born between 37 and 42 weeks of gestation; gestational length; delivery method; live birth rate; birth weight, height and gender.

### Statistical analyses

Results were presented as frequency (percent), median (range) and mean±SD. For parametric data independent samples t-test was used to test differences between groups. For numeric data with nonnormal distribution and ordinal data Mann-Whitney U test was used. Fisher’s exact test was used to test differences between nominal data (frequences).All p values less than 0.05 were considered significant. Statistical data analysis was performed using IBM SPSS Statistics 22 (IBM Corporation, Armonk, NY, USA).

## Results

Study population included 24 pregnant women, 8 (33.3%) in TAI positive study group and 16(66.67%) in TAI negative control group. LT4 supplementation was applied in 5 of 24 patients (20.8%), all of them TAI positive, in order to maintain TSH under 2.5 mIU/mL before IVF treatment. Twin pregnancy was initially confirmed in 10 patients (41.7%). Early miscarriage occured in 2 (8.3%, both TAI negative), late miscarriage in 1 (4.2%, TAI positive), and preterm birth in 4 patients (16.7%, all TAI negative), others (70.8%) gave birth in term. Of 10 twin pregnancies in initial ultrasound control, 4 of them (40%) ended by the birth of two children, and the same number by the birth of one child, while one pregnancy finished by early and other one by late miscarriage. Maternal complications, such as gestational diabetes mellitus or pregnancy induced hypertension, were present in 23.8% (5/21).

We analyzed the outcomes, pregnancy characteristics, hormonal and immunological parameters according to either presence of TAI, or LT4 treatment, and divided patients in the same manner in groups.

Different etiologies, male, female (polycystic ovary syndrome-PCOS, endometriosis, tubal factor), combined male and female, as well as unkown causeof infertility had no impact on pregnancy outcomes, p=0.228 [10].

Characteristics of the study group according to presence of thyroid autoimmunity TAI are listed in Table 1.

**Table 1:** Pregnancy characteristics, hormonal and immunological parameters of the study group according to presence of thyroid autoimmunity. TAI-thyroid autoimmunity referring to the presence of serum thyroid autoantibodies (thyroid peroxidase antibodies and/or thyroglobulin antibodies); TSH-thyrotropin; fT4-free thyroxine; fT3-free triiodothyronine; TPOAbs-thyroperoxidase antibodies; TgAbs-thyroglobulin antibodies; FF-follicular fluid; P4-progesterone. Used statistical tests: aFisher’s exact test, bt-test, cMann–Whitney U test

	TAI positive<br>n=8	TAI negative<br>n=16	p-value
Twin pregnancy, n (%)	2 (25%)	8 (50%)	0.388^a^
Miscarriage, n (%)<br>no<br>early<br>late	<br>7 (87.5%)<br>0 (0.0%)<br>1 (12.5%)	<br>14 (87.5%)<br>2 (12.5%)<br>0 (0.0%)	0.407^a^
Preterm birth, n (%)	0 (0.0%)	4 (28.6%)	0.255^a^
Delivery method, n (%)<br>Vaginal<br>Cesarean section	<br>4 (57.1%)<br>3 (42.9%)	<br>6 (42.9%)<br>8 (57.1%)	0.659^a^
Gestational length<br>(weeks), mean±SD	40.3±1.0	37.4±4.2	0.094^b^
Serum TSH (mIU/mL),<br>mean±SD	1.7±0.5	2.2±0.9	0.200^b^
Serum fT4 (pmol/L),<br>mean±sd	18.5±3.9	17.3±1.9	0.435^b^
Serum fT3 (pmol/L),<br>mean±SD	4.9±0.7	5.5±0.5	0.047^b^
Serum TPOAbs (IU/mL),<br>median (range)	61.7 (8.2–600.0)	11.8 (6.8–25.5)	0.126^c^
Serum TgAbs (IU/mL),<br>median (range)	136.4 (13.7-2380.0)	10.1 (9.0–22.9)	<0.001^c^
FF TSH (mIU/mL),<br>mean±SD	1.7±0.9	1.7±0.8	0.969^b^
FF fT4 (pmol/L),<br>mean±SD	15.9±3.0	15.7±1.9	0.868^b^
FF TPOAbs (IU/mL),<br>median (range)	22.7 (4.0–525.2)	4.0 (4.0-89.8)	0.060^c^
FF TgAbs (IU/mL),<br>median (range)	99.4 (18.1–1488.0)	10.3 (9.0–18.4)	<0.001^c^
FF P4 (ng/mL),<br>median (range)	26100.0 (1500–46950)	21550.0 (11760–47340)	0.759^c^
Birth weight (gr),<br>mean±SD	3578.6±356.9	2758.2±523.4	0.001^b^
Birth height (cm),<br>mean±SD	52.3±2.2	48.7±2.9	0.008^b^

Characteristics of the study group according to presence of LT4 treatment are listed in Table 2.

**Table 2:** Pregnancy characteristics, hormonal and immunological parameters of the study group according to presence of levothyroxine treatment. TAI-thyroid autoimmunity referring to the presence of serum thyroid autoantibodies (thyroid peroxidase antibodies and/or thyroglobulin antibodies); TSH-thyrotropin; fT4-free thyroxine; fT3-free triiodothyronine; TPOAbs-thyroperoxidase antibodies; TgAbs-thyroglobulin antibodies; FF-follicular fluid; P4-progesterone. Used statistical tests: aFisher’s exact test, bt-test, cMann–Whitney U test

	Levothyroxine treatment<br>n=5	No treatment<br>n=19	p-value
Twin pregnancy, n (%)	1 (20.0%)	9 (47.4%)	0.358a
Miscarriage, n (%)<br>no<br>early<br>late	<br>4 (80.0%)<br>0 (0.0%)<br>1 (20.0%)	<br>17 (89.5%)<br>2 (10.5%)<br>0 (0.0%)	0.240^a^
Preterm birth, n (%)	0 (0.0%)	4 (23.5%)	0.546^a^
Delivery method, n (%)<br>Vaginal<br>Caesaream section	<br>4 (100.0%)<br>0 (0.0%)	<br>6 (35.3%)<br>11 (64.7%)	0.035^a^
Gestational length<br>(weeks), mean±SD	40.3±1.3	37.9±3.9	0.307^b^
Serum TSH (mIU/mL),<br>mean±SD	1.7±0.5	2.1±0.9	0.200^b^
Serum fT4 (pmol/L),<br>mean±SD	20.7±2.3	16.9±2.3	0.005^b^
Serum fT3 (pmol/L),<br>mean±SD	4.9±0.6	5.4±0.6	0.124^b^
Serum TPOAbs (IU/mL),<br>median (range)	167.2 (8.2–600.0)	11.7 (6.8–26.6)	0.030c
Serum TgAbs (IU/mL),<br>median (range)	177.4 (13.7–2380.0)	11.2 (9.0-1944.0)	0.010c
FF TSH (mIU/mL),<br>mean±SD	1.5±0.7	1.8±0.8	0.476^b^
FF fT4 (pmol/L),mean±SD	16.6±3.3	15.5±1.9	0.345^b^
FF TPOAbs (IU/mL),<br>median (range)	76.0 (4.0–525.2)	4.0 (4.0–89.8)	0.009c
FF TgAbs (IU/mL),<br>median (range)	143.1 (18.1–1211.0)	10.9 (9.0–1488.0)	0.007c
FF P4 (ng/mL),<br>median (range)	25320.0 (1500–31450)	25750.0 (11760–47340)	0.271^c^
Birth weight (gr),<br>mean±SD	3762.5±327.6	2844.5±530.8	0.001^b^
Birth height (cm),<br>mean±SD	52.3±2.5	49.2±3.1	0.008^b^

Among newborns 11 (45.8%) were male, and 13 (54.2%) female. No congenital malformations in newborns were found. Live birth rate was 96.0% (24/25). Mean birth weight 2997.5±607.4 kg, height 49.7±3.2 cm.

## Discussion

The present study included 24 women with confirmed clinical pregnancy achieved by ART. Live birth rate was 96.0%. Analizing hormonal and immunological parameters it could be noted that TgAbs showed significant difference in serum as well as in FF between the groups according to TAI, while serum fT_3_ was lower in the group with TAI.As was expected, serum fT_4_ was higher in LT4 treated group, with TAI, and newborns in this group had higher birth weight and height. The limitation of the study was small sample size, with a power sample less than 80% for statistically insignificant variables, but it was available number of all consecutively involved patients in the study for the abovementioned period. It is an preliminary study, and further confirmation of the results is needed.

A significant causal association was demonstrated between most preexisting or concomitant diseases presented at the starting time of the first intended oocyte retrieval cycle and lower odds of cumulative live birth [12]. There are meta-analysis emphasizing TAI does not impact IVF outcome in terms of fertilization, implantation and clinical pregnancy, but may have a detrimental effect on the course of a pregnancy, leading to the increased risk of miscarriage and a decreased chance of live birth [13]. The presence of TgAbs is associated with fetal loss even in the absence of thyroid dysfunction [14]. On the other hand, there were studies failed to show any significant differencebetween the anti-TPO+ and anti-TPO− group with respect to live birth delivery, pregnancy or miscarriage, and the same results were presented when comparing subgroups according to TSH level [15].

LT4 supplementation should be recommended to improve clinical pregnancy outcomes in women with thyroid dysfunction, and in infertile women with SCH and/or TAI who are undergoing IVF, mostly to reduce the miscarriage rate [16]. Mature (MII) oocytes from women undergoing IVF contain thyroid hormone (TR) 1, TR 2, TR 1 and TR 2 mRNA, suggesting a possible direct effect of triiodothyronine (T3) on the human oocyte [17]. We demonstrated lower serum fT_3_ in the group with TAI. Hence, T3 is considered a biological amplifier of the stimulatory action of gonadotrophins on granulosa cell function [18].

In a cohort study of 2497 Dutch women without overt thyroid dysfunction, the risk of miscarriage, fetal, and neonatal death increased with higher levels of maternal TSH, i.e. by 60% (OR 1.60, 95% CI[1.04–2.47]) for every doubling in TSH concentration [19]. In large meta-analysis LT4 treatment reduced the miscarriage rate, gestational diabetes, and gestational hypertension, but in the group was fewer preterm deliveries, birth weights <2500 g, deaths and congenital malformations [20]. Two randomized studies evaluated the effect of treatment with LT4 on miscarriage, and reduction in miscarriages with LT4 while other study reported on the effect of LT4 on the rate of preterm birth [21].

Increased likelihood of low birth weight infants born to TPO+ mothers and an increased likelihood of large-for-gestational age infants born to TPO+ women were reported [22]. Carty et al. [23] found that women with a TSH >5 mIU/L delivered lower birth weight babies than those who had a TSH <2.5 mIU/L. Data of the Generation R study demonstrated that in mothers with normal-range fT_4_ and TSH levels, higher maternal fT_4_ levels were associated with lower birth weight, as well as with an increased risk of small size for gestational age at birth newborns [24]. In our study, fT_4_ level was higher in TAI positive group and in the group on LT4 supplementation, but with higher birth weight and height of newborns in these groups.

Treating SCH women remains controversial. Most studies published to date, initiated treatment after the end of the first trimester, which may have been too late to derive benefit, on contrary of those reported an overall reduction in pregnancy complication rate in women treated in the first trimester, but not in those treated in the second trimester [25] [26]. It is worth mentioning treatment of all women in our study was initiated before undergoing ART, thus very early, in order to prepare those women for the procedure, by lowering TSH under 2.5 mmol/L [6]. So, time of the treatment could play a major role. On the other hand, new suggestions for the treatment of thyroid disorders in pregnancy have been proposed [25]. It could be in accordance to Poppe et al. [27] who noted that miscarriage rates in TAI positive women undergoing ART have been declining in contemporary studies compared to in studies reported in the past, due to the increase in rates of ICSI, which may be better option for women with TAI. It could mean that gynecologists get the upper hand in solving the problem.

Although, TPO Abs are more frequently determined in the blood in the sense of TAI, it seems that Tg Abs could play an important role on neonatal outcomes of TAI positive women, and measuring thyroid antibodies in FF in these women could be used to predict the outcomes. Even though, there is still a debate on LT4 treatment pregnancy outcome, a positive effect on neonatal characteristics should not be neglected. Further analysis of thyroid autoantibodies in the FF of women undergoing IVF and their association with adverse pregnancy and neonatal outcomes could help elucidate the mechanisms of TAI impact on the maternal and neonatal complications, all with the aim of achieving a higher birth rate.

In conclusion, thyroid autoantibodies present in FF, in women with TAI undergoing ART, may have an effect on the post-implantation embryo development, but have no effect on further course of pregnancy. Women with TAI and on LT4 supplementation are related to higher newborns weight and height, indicating the special benefit of LT4 treatment for newborn anthropometric parameters.

## Dodatak

### Statement of Ethics

Ethical committee of Faculty of Medicine, University of Belgrade and Ethical committee of Clinic for Gynecology and Obstetrics »Narodni front«, granted approval for the study and written informed consents were obtained from all subjects.

### Funding Sources

This research did not receive any specific grant from any funding agency in the public, commercial or not-for-profit sector.

### Author Contributions

Conceptualization: Sanja Medenica, Eliana Garalejic, Dzihan Abazovic, Milos Zarkovic.

Investigation: Sanja Medenica, Eliana Garalejic, Biljana Arsic, Dzihan Abazovic, Milos Zarkovic.

Methodology: Sanja Medenica, Eliana Garalejic, Zoran Bukumiric, Dzihan Abazovic, Milos Zarkovic.

Resources: Sanja Medenica, Eliana Garalejic, Biljana Arsic, Milos Zarkovic

Supervision: Eliana Garalejic, Stavroula A. Paschou, Snezana Vujosevic, Milos Zarkovic.

Validation: Sanja Medenica, Eliana Garalejic, Dzihan Abazovic, Zoran Bukumiric, Stavroula A. Paschou, Biljana Arsic, Snezana Vujosevic, Biljana Medjo, Milos Zarkovic.

Visualization: Sanja Medenica, Eliana Garalejic, Zoran Bukumiric, Dzihan Abazovic, Biljana Medjo, Milos Zarkovic.

Writing – original draft: Sanja Medenica.

Writing – review & editing: Sanja Medenica, Eliana Garalejic, Stavroula A. Paschou, Milos Zarkovic.

### Conflict of interest statement

All the authors declare that they have no conflict of interest in this work.

### List of abbreviations 

FF, follicular fluid;<br>TAI, thyroid autoimmunity;<br>LT4, levo -thyroxine;<br>ART, assisted reproductive techonology;<br>SCH, subclinical hypothyroidism;<br>IVF, in vitro fertilization;<br>COH, controlled ovarian hyperstimulation;<br>TSH, thyrotropin;<br>ET, embryo transfer;<br>FT4, free thryoxine;<br>FT3, free triiodothyronine;<br>TPOAbs, thyroid peroxidase antibodies;<br>TgAbs, thyroglobulin antibodies;<br>GnRH-a, gonadotropin-releasing hormone agonist;<br>GnRH-ant, gonadotropin-releasing hormone antagonist;<br>hMG, human menopausal gonadotrophin;<br>FSH, follicle-stimulating hormone;<br>hCG, human horionic gonadotropin;<br>E2, estradiol; P4, progesterone;<br>PCOS, polycystic ovary syndrome
